# Optic Flow-Induced Postural and Neuromuscular Responses in Individuals with Type 2 Diabetes over 12 Months: Relationship with Physical Activity Behaviour

**DOI:** 10.3390/biomedicines14061349

**Published:** 2026-06-15

**Authors:** Alessandra Laffi, Alessandro Piras, Andrea Meoni, Lucia Brodosi, Federica Perazza, Maria Letizia Petroni, Milena Raffi

**Affiliations:** 1 Department of Biomedical and Neuromotor Sciences DIBINEM, University of Bologna, 40126 Bologna, Italy; alessandra.laffi3@unibo.it (A.L.); andrea.meoni@unibo.it (A.M.); 2 Department of Quality of Life Studies QUVI, University of Bologna, 47921 Rimini, Italy; alessandro.piras3@unibo.it; 3 Clinical Nutrition and Metabolism Unit, IRCCS AOUBO, 40138 Bologna, Italy; lucia.brodosi2@unibo.it; 4 Department of Medical and Surgical Sciences, University of Bologna, 40138 Bologna, Italy; federica.perazza2@unibo.it (F.P.); marialetizia.petroni@unibo.it (M.L.P.)

**Keywords:** EMG, COP displacement, body sway, optic flow, postural control, balance control, heading perception, diabetes, muscle activity

## Abstract

**Background:** Exercise plays a crucial role in the prevention and management of type 2 diabetes. During self-motion, optic flow provides visual information about heading direction and influences postural control. This study investigated postural responses and muscle activation in individuals with type 2 diabetes exposed to optic flow stimuli simulating self-motion, and examined whether these responses varied according to habitual physical activity over 12 months. **Methods:** Surface electromyographic (EMG) and stabilometric data were collected from 23 individuals during quiet standing under different visual motion conditions. Participants were classified as physically active or inactive based on standardized criteria. EMG activity was recorded bilaterally from the tibialis anterior and soleus muscles at baseline, 6, and 12 months. Center of pressure (COP) displacement was measured using two force platforms. **Results:** Stabilometric analysis revealed a significant effect of visual stimulus on COP displacement in both antero-posterior and medio-lateral directions, as well as on COP speed, indicating that optic flow modulates postural control. COP speed changes over time differed by sex, while medio-lateral sway showed time-dependent variations across sides and physical activity groups. EMG analysis showed a significant effect of visual stimulus on soleus activation, with no consistent effects for tibialis anterior. **Conclusions:** Optic flow significantly modulated postural control and lower-limb muscle activation in individuals with type 2 diabetes. Preliminary differences in response profiles associated with habitual physical activity level were observed, though these should be interpreted cautiously given the exploratory nature of the study. Larger, adequately powered studies are warranted to further investigate these associations.

## 1. Introduction

Type 2 diabetes mellitus (T2DM) is a chronic metabolic disease characterized by persistent hyperglycemia, primarily driven by insulin resistance and accompanied by dyslipidemia and low-grade systemic inflammation [1]. These metabolic disturbances promote progressive organ damage, leading to both microvascular complications, such as diabetic retinopathy, nephropathy, and neuropathy, and macrovascular complications, including ischemic heart disease, stroke, and peripheral arterial disease, making T2DM a leading cause of morbidity and mortality worldwide [2].

Regular physical activity is a cornerstone of T2DM management and is strongly recommended by international clinical guidelines [3]. Aerobic exercise, in particular, is consistently identified as the first-line and most accessible modality for individuals with T2DM, owing to its well-documented benefits on cardiorespiratory fitness, insulin sensitivity, and glycaemic control, as well as its feasibility across a wide range of functional capacities [4]. The World Health Organization recommends that adults engage in at least 150–300 min per week of moderate-intensity aerobic physical activity, or 75–150 min per week of vigorous-intensity activity, or an equivalent combination, as a minimum threshold for health benefits. Failure to meet these recommendations is associated with increased cardiometabolic risk and is highly prevalent among individuals with T2DM [5]. Despite clear recommendations, long-term engagement in structured physical activity remains limited in people with T2DM. Behavioural barriers, excess body weight, musculoskeletal discomfort, and reduced mobility frequently hinder sustained participation, particularly in populations with obesity or functional limitations. For these reasons, low-threshold aerobic exercise programmes delivered in structured yet adaptable formats are considered especially relevant in real-world clinical settings [6]. In this context, both land-based and aquatic aerobic exercise modalities have been shown to be safe and effective for individuals with T2DM. Aquatic aerobic training, in particular, may enhance adherence and feasibility in individuals with overweight, obesity, or joint discomfort, while still eliciting improvements in glycaemic control, cardiovascular risk factors, and functional outcomes [7].

Beyond metabolic outcomes, T2DM is increasingly recognized as a condition associated with impairments in motor function and postural control [8]. Balance regulation depends on the integration of visual, vestibular, and somatosensory inputs and on the coordinated activation of postural muscles. In individuals with T2DM, this integration may be compromised by peripheral neuropathy, visual dysfunction, and altered sensory processing, leading to increased postural sway and a higher risk of falls [8].

Visual information plays a central role in postural control through optic flow, defined as the pattern of visual motion generated during self-motion. Optic flow stimulation is an established experimental approach for investigating the contribution of visual information to postural control, as it enables controlled manipulation of visual motion cues that influence body sway and postural muscle activity [9,10,11]. Experimental studies have demonstrated that optic flow significantly modulates centre-of-pressure dynamics and lower-limb muscle activation during upright stance [9,12,13]. In individuals with diabetes-related visual impairment, such as diabetic retinopathy, altered processing of optic flow has been associated with enhanced postural responses and compensatory neuromuscular strategies [14].

In individuals with T2DM, who may exhibit subtle alterations in visual processing, somatosensory feedback, or central integration, the exposure to dynamic visual environments may offer additional insights into postural regulation beyond those obtained under static conditions [14]. In recent years, increasing interest has been directed toward understanding balance control and sensory-motor integration in people with T2DM, particularly in relation to fall risk and functional performance [8,14,15]. Further, few studies have examined the role of physical activity on balance control in individuals with T2DM [16,17,18]; however, it remains unclear whether habitual physical activity level influences stabilometric and neuromuscular responses to optic flow stimulation in individuals with T2DM over time. Previous research in this population has predominantly focused on metabolic and general functional outcomes (see [3] for review) [4], while the relationship between physical activity engagement, dynamic visual processing, and postural control has received comparatively less attention.

Within this expanding research context, it remains of interest to examine whether sustained engagement in aerobic physical activity, defined according to standardized physical activity thresholds, is associated with differences in stabilometric and electromyographic responses during optic flow exposure. Exploring these associations may contribute to a more comprehensive understanding of the functional adaptations related to physical activity in individuals with T2DM. Accordingly, the aim of the present study was to examine whether postural control and lower-limb muscle activation in individuals with T2DM differed according to habitual physical activity classification over a 12-month period. Stabilometric and surface electromyographic measures were used to characterize balance-related and neuromuscular responses under visually challenging conditions. The present study adds longitudinal data on optic flow-related postural and neuromuscular responses in individuals with T2DM in relation to habitual physical activity behaviour, extending previous research beyond predominantly metabolic outcomes.

## 2. Materials and Methods

### 2.1. Participants

The study involved 23 individuals with type 2 diabetes. Participants were classified as physically active or physically inactive according to standardized physical activity criteria [5] that are described in detail in Section 2.2. The active group included 13 diabetic people (age 57.5 ± 10.4 years; BMI 29.5 ± 5.2), while the inactive group was formed by 10 diabetic people with sedentary habits (age 64.4 ± 5.2 years; BMI 33.0 ± 5.6). Between-group comparisons confirmed statistical comparability at baseline, with no significant differences detected for any variable at α = 0.05 (Table 1). Given the limited sample size, this study should be considered exploratory, and the findings are intended to be hypothesis-generating.

All participants had a diagnosis of established T2DM with stable metabolic control and were under routine outpatient care at the recruitment site, receiving stable pharmacological treatment throughout the study period, including oral hypoglycaemic agents and/or insulin therapy. No episodes of acute glycemic decompensation or changes in diabetes medication were reported during follow-up. Eligibility and suitability for safe participation were confirmed through clinical screening, which included neurological examination and review of medical history conducted by the referring physician as part of routine care. None of the participants had a diagnosis of peripheral neuropathy, physical deficit, or muscular injury at the time of enrolment. Diabetes duration was categorized as <5 years (*n* = 8) or ≥5 years (*n* = 15); the two subgroups did not differ at baseline for age, anthropometric, or glycaemic variables (all *p* > 0.05), with the exception of height (*p* = 0.026), reflecting the higher proportion of males in the shorter-duration subgroup (Table 1). Diabetes duration was evenly distributed across the physically active and sedentary groups (Fisher’s exact test, *p* = 1.000). All subjects had normal or corrected-to-normal vision. No economic compensation was provided to participants.

The study was approved by the Bioethics Committee of the University of Bologna, protocol no. 0283851 (4 November 2021) and protocol no. 0351417 (7 November 2024). Each participant read and signed the informed consent to participate in the study. The experiments were performed in accordance with the ethical standards laid down in the 1964 Declaration of Helsinki.

The hand and foot laterality of each subject was assessed by the standardized Waterloo Footedness Questionnaire (WFQ) and Waterloo Handedness Questionnaire (WHQ) [19] with the following formula:

$$LI=\frac{\left(Right\;preference-Left\;preference\right)}{\left(Right\;preference+Left\;preference\right)}\times 100$$

A positive laterality index (LI) indicates a right dominance, while a negative index indicates a left dominance.

### 2.2. Physical Activity Assessment and Classification

At baseline, all participants reported no engagement in structured physical activity. To promote physical exercise engagement and to address common barriers frequently observed in individuals with type 2 diabetes [4,20], participants were offered access to a standardized, low-threshold aerobic exercise program lasting 12 months.

Training sessions consisted of aerobic exercise and were delivered either as supervised land-based aerobic circuit training in a gym or as structured aquatic aerobic cardio-circuit sessions in a swimming pool. The choice of setting was based on individual feasibility considerations, such as overweight or obesity, joint discomfort, or reduced mobility [7,21]. Exercise modality (aerobic training) was consistent across participants, while the exercise setting, land-based or aquatic, was self-selected and remained unchanged throughout the follow-up period.

Physical activity behaviour was assessed at 6- and 12-month follow-up through a structured face-to-face interview conducted by trained researchers. Participants were asked to report, for the previous seven days, the type of physical activity performed, the duration of each session (minutes), and the weekly frequency (days/week). The self-reported activities were then categorised and quantified according to the data processing and scoring procedures defined by the International Physical Activity Questionnaire (IPAQ) Research Committee [22]. Each reported activity was assigned a metabolic equivalent of task (MET) value based on the Adult Compendium of Physical Activities [23]. For classification purposes only, MET-min/week values were calculated by averaging the 6- and 12-month assessments. Weekly energy expenditure was calculated following the standard procedure indicated in the IPAQ guidelines:

MET-min/week = MET value × minutes of activity × days per week.

All participants were classified as physically inactive at baseline; group classification therefore emerged over the course of the study rather than reflecting a pre-existing difference between individuals. Participants’ engagement in the exercise programme was voluntary and self-selected rather than assigned by investigators. Physical activity measures were used exclusively for group classification and were not included as longitudinal outcomes in the statistical analysis. Observed differences in postural and neuromuscular response patterns between groups may therefore reflect the cumulative effects of differential engagement with the offered programme during follow-up, rather than the influence of long-standing habitual physical activity behaviour. Accordingly, between-group comparisons should be interpreted in the context of this emergent classification, and causal inferences regarding the role of habitual physical activity remain unwarranted.

### 2.3. Optic Flow Stimuli

The visual stimuli used in this study were identical to those previously employed by Piras et al. [14] to investigate postural responses to optic flow in individuals with diabetic retinopathy. These stimuli were selected to investigate distinct spatial and directional components of optic flow processing relevant to postural control. The selected dot speed (5°/s) was intended to induce measurable postural perturbation while remaining consistent with previous optic flow studies [9,12,14]. Peripheral retinal flow is considered a major contributor to automatic postural responses, whereas foveal stimulation is believed to serve as a contrasting condition with reduced directional motion information per unit retinal area (see [24] for review). Central expanding optic flow simulates forward self-motion and is known to elicit robust postural responses [13,14]. Together, these conditions were used to explore visuomotor integration mechanisms potentially involved in balance regulation in individuals with T2DM, a population in which subtle alterations in sensory integration and visual processing have previously been reported [8].

The stimuli consisted of white radial dots (luminance: 1.3 cd/m^2^; diameter: 0.4°), projected onto a translucent screen covering 135° × 107° of the visual field. The experimental setup was arranged in a dark room, with participants standing barefoot on dual force platforms, instructed to fixate on a central point projected on the screen. The fixation point was individually adjusted to align with each participant’s eye level. The screen was positioned at a distance of 115 cm from the participants’ eyes.

**Figure 1:**
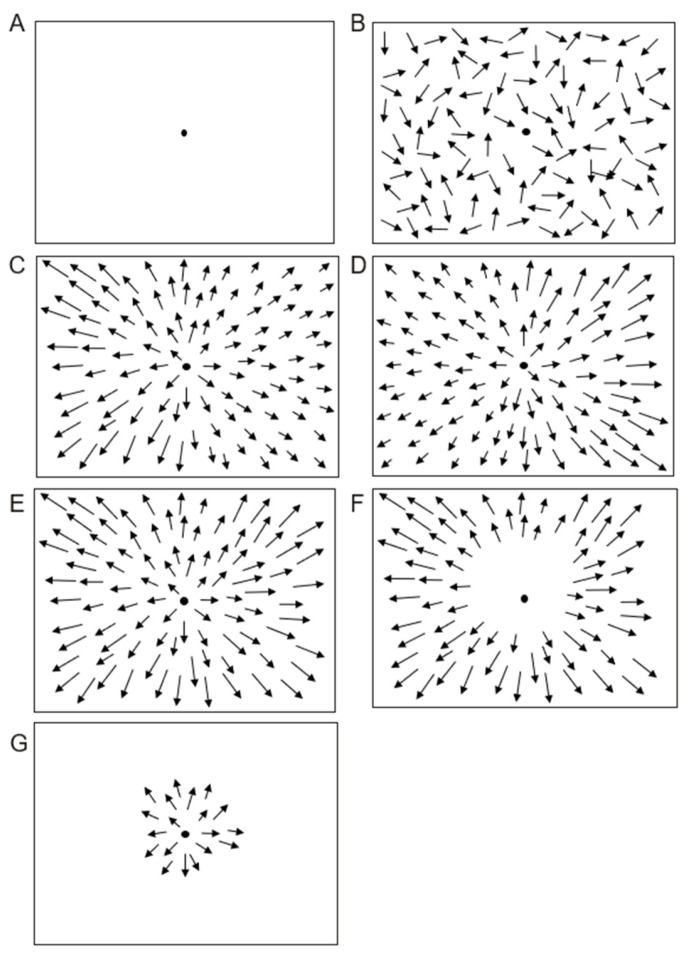
Optic flow stimuli. (**A**) Fixation on a dark screen (Baseline). (**B**) Random dots motion (Random). (**C**) Fixation point (FP) to the centre and dots accelerated to the left simulated heading to the left with fixation straight ahead (OF-L). (**D**) FP to the centre and dots accelerated to the right simulated heading to the right with fixation straight ahead (OF-R). (**E**) Full field radial expansion with the FP simulated heading and fixation straight ahead (OF-C). (**F**) Peripheral stimulation, the blank area in the centre had a radius of 20° (Periphery). (**G**) Foveal stimulation, the stimulated area had a radius of 7° (Fovea). Arrows represent the velocity and direction vectors of moving dots.

We randomly presented seven optic flow stimuli (Figure 1). The randomization was identical for all subjects to ensure identical stimulus presentation across participants, without involving any potential biases or judgments. Fixation on a dark screen (Figure 1A, Baseline) and a full-field stimulus composed of randomly moving dots (Figure 1B, Random) were used as control stimuli. We used two types of optic flow motion to simulate different headings: in the first condition, the dots’ speed accelerated to the left to simulate left-heading direction (Figure 1C, OF-L), while in the second condition, the speed accelerated to the right to simulate right-heading direction (Figure 1D, OF-R). Three expanding optic flow stimuli were presented full field (Figure 1E, OF-C), in the peripheral region (Figure 1F, Periphery; the blank area in the centre had a radius of 20°) and in the foveal region (Figure 1G, Fovea) the stimulated area had a radius of 7°. The fixation point was placed in the center of the screen. Participants were instructed to maintain fixation for the entire trial duration. Optic flow stimuli were made using MATLAB Psychophysics Toolbox version 2 (The Mathworks Inc., Natick, MA, USA). For each stimulus, we recorded two trials lasting 30 s.

### 2.4. Stabilometric and Electromyographic Recordings

Experiments were performed in a quiet room with stable temperature (21 °C; 52% of humidity). Subjects were asked to avoid drinking caffeinated beverages before the experimental procedures and were instructed to avoid strenuous activity and alcohol in the 12 h preceding the test.

Stabilometric data were collected at a sampling rate of 1000 Hz using two adjacent force platforms (Kistler^®^, Kistler Instrument Corp., Milan, Italy), allowing separate recording of ground reaction forces under each foot. Participants stood barefoot on the force platforms with feet aligned to a standardized reference line marked for the halluces, arms relaxed at their sides, and gaze directed toward the central fixation point.

Electromyographic data were acquired at 1000 Hz using a wireless PocketEMG system (FREE1000 EMG—BTS Bioengineering Inc., Garbagnate Milanese, Italy). Disposable Ag/AgCl electrodes (32 × 32 mm; RAM Apparecchi Medicali s.r.l., Genova, Italy), with an active area of 0.8 cm^2^ and an inter-electrode distance of approximately 2 cm, were applied to shaved and cleaned skin to reduce impedance. Electrodes were placed bilaterally over the muscle belly of the tibialis anterior (left: LTA; right: RTA) and soleus (left: LSOL; right: RSOL), following SENIAM guidelines.

### 2.5. Postural and Neuromuscular Data Processing and Statistical Analysis

EMG signals were positively rectified and band-pass filtered (Butterworth filter, 20–450 Hz) using SMART Analyzer (BTS Bioengineering Inc.). Each trial was normalized to the mean muscle activation recorded during the baseline condition (static fixation on a dark background). Normalized root mean square (RMS) values were calculated in 100 ms bins using MATLAB 2024a (The MathWorks Inc., Natick, MA, USA). Repeated-measures ANOVA was performed on normalized EMG signals to assess the effects of physical activity over time. Time (baseline, 6 months, 12 months), stimulus (six visual optic flow conditions), and side (right, left) were included as within-subject factors, while group (active, sedentary) and sex (male, female) were included as between-subject factors. For EMG analysis, muscle activity recorded during the baseline condition (static fixation on a dark screen) was used exclusively as a reference for signal normalization and was therefore not included as a level of the stimulus factor. Accordingly, the definition of the stimulus factor differed between stabilometric and electromyographic analyses, reflecting the different roles of the baseline condition in the two signal processing approaches.

Stabilometric data were low-pass filtered at 15 Hz. Antero-posterior (AP) and medio-lateral (ML) components of the center of pressure (COP), as well as COP area and COP speed, were analyzed separately using repeated-measures ANOVA. For all stabilometric variables, stimulus (seven visual conditions), side (right, left), and time (baseline, 6 and 12 months) were included as within-subject factors, while group (active, sedentary) and sex (male, female) were included as between-subject factors.

All statistical analyses were performed using IBM^®^ SPSS^®^ Statistics for Windows (version 27.0; IBM Corp., Armonk, NY, USA). Sphericity was assessed using Mauchly’s test, and when the assumption was violated (*p* < 0.05), Greenhouse–Geisser correction was applied. Effect sizes were calculated using partial eta squared (ηp^2^), and statistical significance was set at *p* < 0.05. No formal correction for multiple comparisons was applied across ANOVA models, consistent with the exploratory nature of the study. No missing data were present in the stabilometric or electromyographic datasets; therefore, all repeated-measures analyses were conducted on complete dataset.

### 2.6. Anthropometric and Biochemical Data Analysis

Anthropometric and biochemical parameters were collected concurrently with the experimental sessions to characterize the clinical profile of the participants and to assess potential metabolic changes throughout the follow-up period. Body weight, body mass index (BMI), abdominal circumference, glycated hemoglobin (HbA1c), fasting glucose, total cholesterol, high-density lipoprotein (HDL), low-density lipoprotein (LDL), and triglycerides were analysed using non-parametric tests for repeated measures. Specifically, the Friedman test was employed to evaluate changes across time points (baseline, 6 months, and 12 months), owing to non-normal data distributions and the presence of missing values in several variables. When the Friedman test yielded significant results, post hoc pairwise comparisons were performed using Wilcoxon signed-rank tests with Bonferroni correction. All statistical analyses were conducted using IBM^®^ SPSS^®^ Statistics for Windows (version 27.0; IBM Corp., Armonk, NY, USA).

## 3. Results

**Figure 2:**
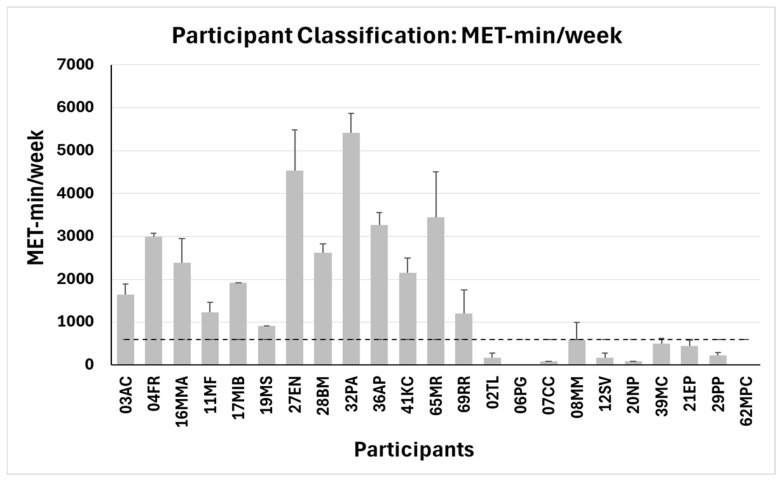
Weekly energy expenditure (MET-min/week) per participant (mean ± SD, averaged across 6- and 12-month assessments). Dashed line indicates the IPAQ cut-off (600 MET-min/week) used for classification [22,25].

Baseline anthropometric and metabolic characteristics of the participants are reported in Table 1. Physical activity levels used to classify participants into active and sedentary groups [22,25], expressed as mean ± SD of the 6- and 12-month assessments, are shown in Figure 2.

Laterality assessment revealed a predominantly right-lateralized profile. Lateralization index (LI) ranged from 14.29 to 100, with 80% of participants showing strong right dominance (LI ≥ 80). The remaining participants exhibited moderate right lateralization. No left-oriented dominance was observed.

### 3.1. Stabilometric Parameters

**Figure 3:**
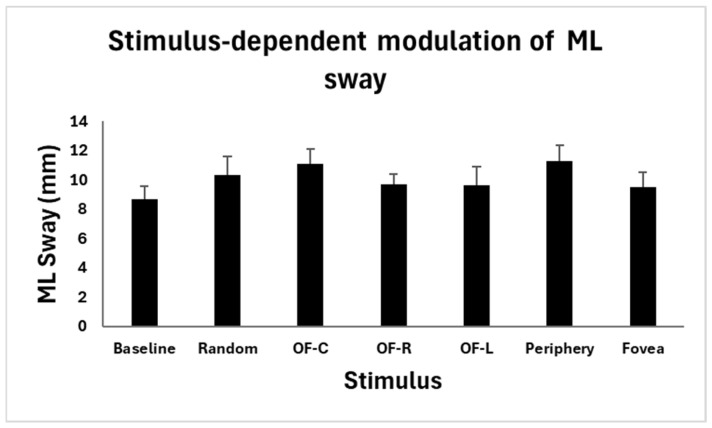
Medio-lateral COP sway across visual stimulus conditions. Bars represent mean ± standard error (SE). Data were analysed using repeated-measures ANOVA with stimulus, side, and time as within-subject factors and group and sex as between-subject factors.

**Figure 4:**
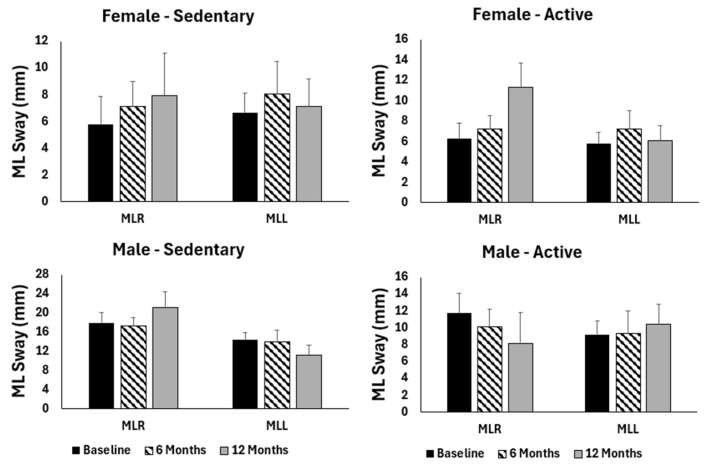
Time-, sex-, and group-dependent changes in medio-lateral center of pressure (COP) sway. Separate panels show females and males in active and sedentary groups. For each condition, bars represent medio-lateral sway for the right (MLR) and left (MLL) sides at baseline, 6 months, and 12 months. Data are presented as mean ± standard error. Data were analysed using repeated-measures ANOVA with stimulus, side, and time as within-subject factors and group and sex as between-subject factors.

For medio-lateral (ML) COP displacement, a significant main effect of stimulus was observed (F = 2.934, *p* = 0.045, ηp^2^ = 0.557), with lowest values during baseline fixation and highest values during full-field and peripheral stimulation (Figure 3). Figure 4 shows a significant side × time × group × sex interaction (F = 4.430, *p* = 0.027, ηp^2^ = 0.330). Given the exploratory nature of the analyses and the absence of correction for multiple comparisons, these findings should be considered preliminary and require replication in studies with larger sample sizes. Higher ML COP displacement was observed in males compared to females across groups, with more pronounced side- and time-related variations in males (Figure 4).

**Figure 5:**
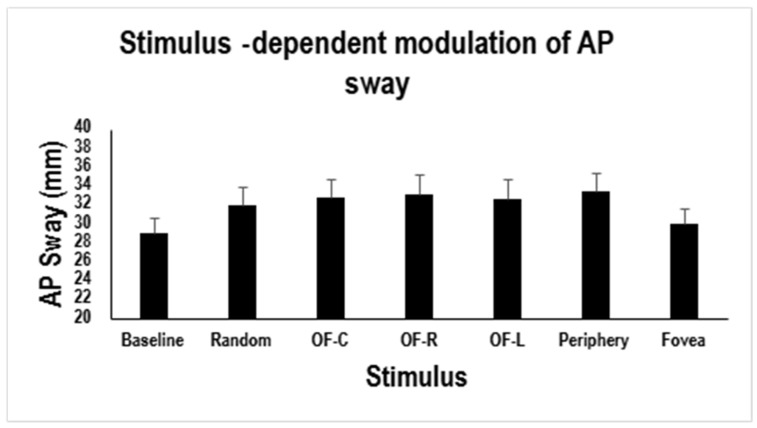
Antero-posterior COP sway across visual stimulus conditions. Data are reported as mean ± standard error. Data were analysed using repeated-measures ANOVA with stimulus, side, and time as within-subject factors and group and sex as between-subject factors.

For the antero-posterior (AP) component of COP displacement, a significant main effect of stimulus was observed (*F* = 3.47, *p* = 0.026, ηp^2^ = 0.598), with increased displacement during visual motion conditions compared to baseline fixation (Figure 5).

**Figure 6:**
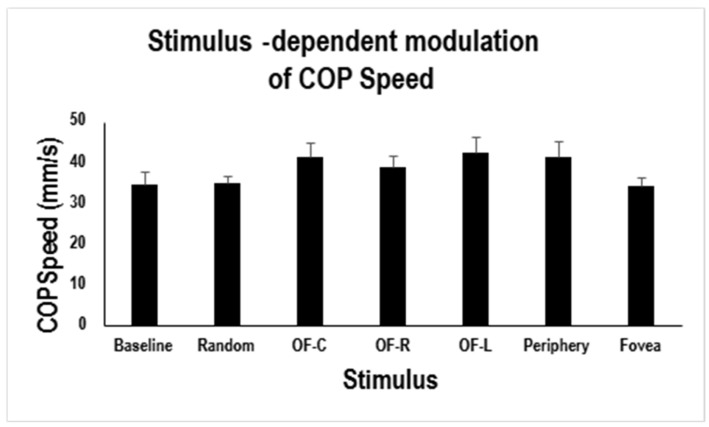
Stimulus-dependent modulation of COP speed across visual conditions. Data are reported as mean ± SE. Data were analysed using repeated-measures ANOVA with stimulus, side, and time as within-subject factors and group and sex as between-subject factors.

**Figure 7:**
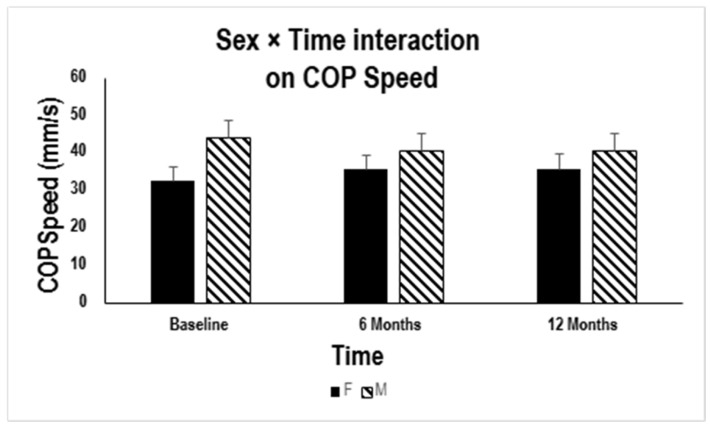
Sex × time interaction on COP speed at baseline, 6 months, and 12 months. Data are reported as mean ± SE. Data were analysed using repeated-measures ANOVA with stimulus, side and time as within-subject factors and group and sex as between-subject factors.

COP speed showed a significant main effect of stimulus (*p* < 0.001). Multivariate analysis revealed marked differences in COP speed across visual conditions (*F* = 26.43, *p* < 0.001, ηp^2^ = 0.919). As shown in Figure 6, COP speed was higher during optic flow conditions (central, right, left, and peripheral) compared with baseline, random, and foveal stimulation. A significant time × sex interaction was also observed (*F* = 4.05, *p* = 0.035, ηp^2^ = 0.311); as illustrated in Figure 7, males exhibited consistently higher COP speed than females at all time points, while females showed a modest increase over time, resulting in a convergence of values at the 12-month follow-up.

### 3.2. Electromyographic Activity

**Figure 8:**
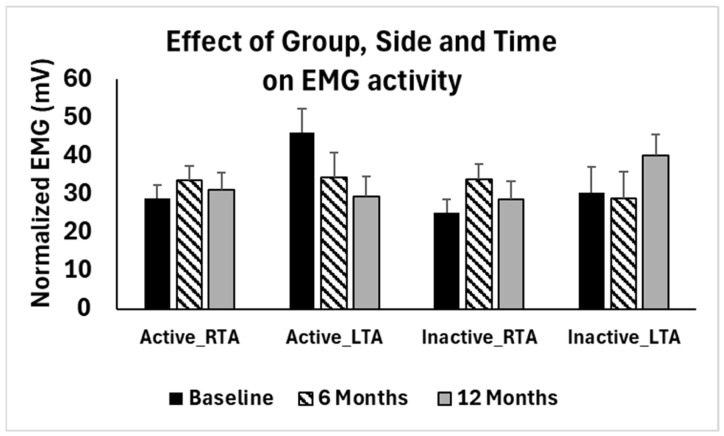
Normalized tibialis anterior (TA) EMG activity across groups (active vs. inactive), sides (right, left) and time points (baseline, 6 and 12 months). Data are presented as mean ± SE. Data were analysed using repeated-measures ANOVA with stimulus, side, and time as within-subject factors and group and sex as between-subject factors.

**Figure 9:**
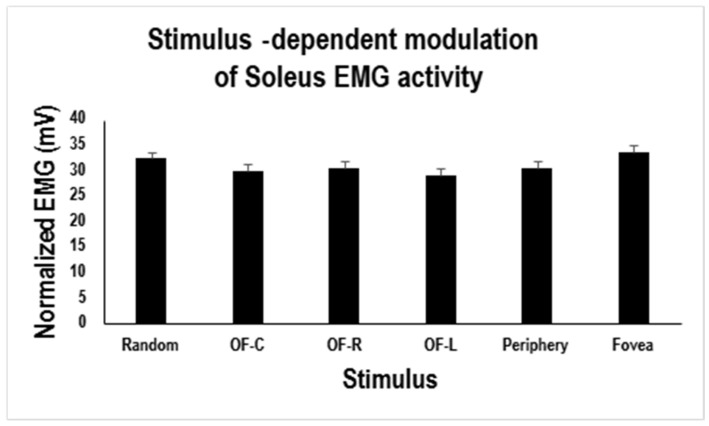
Normalized soleus EMG activity across visual stimulus conditions. Data are reported as mean ± standard error. Data were analysed using repeated-measures ANOVA with stimulus, side, and time as within-subject factors and group and sex as between-subject factors.

For the tibialis anterior muscle, a side × time × group interaction reached the conventional significance threshold (F = 3.55, *p* = 0.050, ηp^2^ = 0.283), with higher baseline EMG activity in the active group and a tendency toward increased activation over time in the inactive group, particularly on the left side (Figure 8). Although the present findings suggest a potential association, further studies with larger samples are needed to establish the robustness and generalizability of these observations. A significant main effect of stimulus was observed for soleus EMG activity (F = 9.16, *p* < 0.001, ηp^2^ = 0.753), with higher normalized activation during the foveal and random conditions compared to the central, rightward, leftward and peripheral optic flow conditions (Figure 9).

### 3.3. Anthropometric and Biochemical Parameters

A significant effect of time was observed for total cholesterol (χ^2^(2) = 8.51, *p* = 0.014). Post hoc pairwise comparisons with Bonferroni correction revealed a significant reduction at 12 months compared with baseline (*p* = 0.013), while no differences were found between baseline and 6 months or between 6 and 12 months. For LDL cholesterol, Friedman analysis revealed a significant effect of time (χ^2^(2) = 10.34, *p* = 0.006). Post hoc Wilcoxon tests showed significantly lower LDL levels at both 6 months (*p* = 0.018) and 12 months (*p* = 0.018) compared with baseline, with no significant difference between 6 and 12 months. These results indicate that, over the follow-up period, lipid profile variables showed selective time-dependent changes, while glycaemic control and anthropometric measures remained stable. No significant changes across time were observed for body weight (χ^2^(2) = 0.75, *p* = 0.688), body mass index (χ^2^(2) = 0.75, *p* = 0.688), abdominal circumference (χ^2^(2) = 0.16, *p* = 0.923), HbA1c (χ^2^(2) = 4.35, *p* = 0.113), fasting glucose (χ^2^(2) = 5.12, *p* = 0.077), HDL cholesterol (χ^2^(2) = 2.91, *p* = 0.233), or triglycerides (χ^2^(2) = 2.09, *p* = 0.353). As no formal correction for multiple comparisons was applied across biochemical outcomes, these results should be considered hypothesis-generating and warrant confirmation in future studies.

## 4. Discussion

This study examined whether long-term habitual physical activity level was associated with differences in postural control and neuromuscular activation during optic flow stimulation in individuals with T2DM. The primary finding was a robust stimulus-dependent modulation of COP dynamics and soleus EMG activity, confirming that dynamic visual stimuli engage visuomotor integration mechanisms relevant to balance control even in the absence of mechanical perturbations. Group-dependent effects were not observed as main effects, but emerged as components of higher-order interactions, consistent with the exploratory nature of the study and the sample size. The summary of results is shown in Table 2. Together, these findings situate optic flow stimulation as a sensitive paradigm for probing sensorimotor function in a population characterised by subtle impairments in sensory integration and postural control.

### 4.1. Stabilometry

Antero-posterior (AP) sway exhibited a relatively uniform increase in response to visual motion across experimental conditions, consistent with models describing AP postural control as predominantly governed by ankle-based strategies. In this framework, the body behaves as a single-link inverted pendulum responding to sensory perturbations through modulation of ankle musculotendinous stiffness [12,13]. Accordingly, the presence of dynamic visual input alone appears sufficient to elicit increased AP sway, largely independent of the spatial characteristics or direction of the stimulus.

In contrast, medio-lateral (ML) COP displacement showed a more complex and stimulus-specific modulation, characterized by significant interactions involving body side, time, group, and sex. ML stability is considered an actively regulated component of balance and is, therefore, more sensitive to sensory integration demands [15]. Present results regarding the greater ML sway observed under full-field and peripheral optic flow conditions support the dominant contribution of peripheral vision to automatic postural regulation [9,12,13]. This interpretation is consistent with evidence from individuals with diabetic retinopathy, in whom impaired peripheral retinal processing compromises optic flow integration, leading to increased COP oscillations and less adaptable neuromuscular strategies [14]. The side- and sex-dependent temporal patterns observed in this predominantly right-dominant cohort further suggest that ML control reflects lateralized neuromotor strategies rather than purely biomechanical constraints. These findings align with evidence indicating that instability in the medio-lateral direction is particularly sensitive to adaptive balance control mechanisms and may be clinically relevant in populations at increased risk of falls, including individuals with type 2 diabetes [15,26].

COP velocity has been proposed not as a direct marker of postural instability, but rather as an index of the intensity and frequency of active postural corrections generated by the neuromotor system [27]. In the present study, the dissociation between increased COP velocity and unchanged sway area suggests a pattern of more frequent corrective adjustments without a concomitant expansion of spatial sway. This pattern may reflect more frequent corrective postural adjustments under dynamic visual conditions, consistent with adaptive sensorimotor regulation during optic flow exposure [12,13], although alternative explanations cannot be excluded. At the same time, previous studies in older and diabetic populations have suggested that increased COP velocity, particularly in the absence of larger sway amplitudes, may also reflect reduced postural control efficiency or increased sensorimotor demand [8,20]. Within this framework, the present findings may suggest altered visuomotor regulation under dynamic visual stimulation, although the relative contribution of adaptive versus less efficient control mechanisms cannot be fully determined from the present data.

Regardless of the underlying mechanism, the observed pattern may be compatible with models of multisensory integration and sensory reweighting, whereby the central nervous system dynamically adjusts the relative contribution of sensory inputs based on their reliability. When optic flow provides coherent and informative visual cues, visual input may gain greater weight in estimating body orientation, allowing reduced reliance on tonic peripheral stabilization mechanisms and promoting more flexible control strategies [9,13,14]. In the present study, the increase in COP velocity in the absence of substantial changes in sway area may therefore reflect a visually guided control strategy characterized by continuous, small-amplitude adjustments rather than mechanical stiffening of the postural system.

The significant Sex × Time interaction observed for COP velocity further indicates that postural control dynamics evolved across repeated assessments. While males exhibited consistently higher COP velocity values, females showed a modest but progressive increase over time, resulting in convergence at the 12-month follow-up. This temporal pattern may suggest changes in postural control strategies across repeated assessments, although the specific mechanisms underlying this adaptation remain to be elucidated.

### 4.2. Electromyography

Electromyographic findings support the interpretation of active postural regulation. Reduced soleus activation during structured optic flow stimulation, despite increased COP dynamics, could indicate decreased reliance on tonic ankle stabilization when visual information is coherent. Conversely, higher soleus activity during foveal and random visual conditions likely reflects compensatory increases in peripheral control when visual cues are less reliable. The absence of robust main effects for tibialis anterior activity indicates that this muscle may primarily contribute to rapid, context-dependent corrective responses rather than sustained postural support. Overall, these findings indicate that optic flow influences postural control in individuals with type 2 diabetes primarily through modulation of neuromuscular recruitment patterns rather than through large changes in sway amplitude.

In individuals with T2DM, sensorimotor pathways involved in postural regulation may be partially compromised even in the absence of clinically diagnosed neuropathy [8]. The observed differences in COP dynamics and muscle activity between physically active and sedentary participants are noteworthy, particularly given that glycaemic control and anthropometric measures remained largely stable and comparable across groups throughout the follow-up period. This pattern suggests that the postural and neuromuscular differences identified here are unlikely to be primarily driven by metabolic factors, given the relative stability of glycaemic and anthropometric measures throughout follow-up. Rather, these findings may be compatible with differences in sensorimotor function associated with physical activity engagement. Such an interpretation is consistent with previous evidence indicating that exercise-related improvements in balance and postural control in T2DM may occur independently of changes in glycaemic indices [21]. Taken together, these findings raise the possibility that physical activity behaviour may be associated with differences in visuomotor and postural responses in individuals with T2DM, a relationship that warrants further investigation in larger longitudinal studies.

### 4.3. Strengths and Limitations

This study provides a detailed characterisation of postural and neuromuscular responses to optic flow stimulation in individuals with T2DM by integrating stabilometric and electromyographic measures within a controlled experimental paradigm. Dynamic visual stimulation elicited coherent modulation of postural dynamics and muscle activation, supporting the robustness of the main stimulus-related findings.

Several limitations should be considered. The study was exploratory in nature, and the sample size was not based on an a priori power calculation; consequently, small effects, particularly higher-order interactions, may have gone undetected. Although the principal findings were associated with moderate-to-large effect sizes, interaction effects should be considered preliminary. In addition, no formal correction for multiple comparisons was applied, increasing the possibility of Type I error, particularly for interactions with *p*-values close to the significance threshold. These findings should therefore be regarded as hypothesis-generating and require confirmation in larger, adequately powered cohorts.

Physical activity was assessed through self-report, and participants self-selected both their engagement with the exercise programme and the exercise setting, introducing the possibility of self-selection bias. Accordingly, residual confounding related to motivation, functional capacity, baseline health status, age, BMI, or other unmeasured characteristics cannot be completely excluded, and causal interpretations are not warranted.

The absence of a non-diabetic control group limits inference regarding diabetes-specific postural profiles. Furthermore, peripheral neuropathy and retinopathy were excluded on the basis of routine clinical assessment rather than instrumental screening, and diabetes duration was available only as a categorical variable. Future studies employing larger samples, objective physical activity monitoring, instrumental clinical assessments, and longitudinal or interventional designs are needed to clarify the mechanisms underlying the associations observed here.

## 5. Conclusions

In individuals with T2DM, exposure to optic flow stimuli significantly modulated postural control and lower-limb muscle activation, with stimulus-dependent effects on COP displacement and soleus EMG activity. These findings confirm the central role of visuomotor integration in balance regulation in this population and highlight the sensitivity of stabilometric and electromyographic measures to dynamic visual stimulation. Complex time-, side-, and sex-dependent patterns were observed in stabilometric and neuromuscular responses across the 12-month follow-up, in the context of largely stable glycaemic and anthropometric parameters. Preliminary differences in response profiles between participants with different habitual physical activity levels were also identified, though these should be interpreted cautiously given the exploratory nature of the analyses and the sample size. Taken together, these findings contribute to a growing body of evidence on sensorimotor function in T2DM and suggest that the relationship between habitual physical activity and visuomotor postural regulation warrants further investigation in larger, adequately powered studies.

## Figures and Tables

**Table 1:** Baseline characteristics of study participants. Values are expressed as mean ± standard deviation (SD). Between-group differences were assessed using one-way ANOVA for continuous variables and Fisher’s exact test for the categorical variable (sex). No statistically significant differences were observed between groups at baseline (*p* > 0.05). BMI, body mass index; HbA1c, glycated hemoglobin (mmol/mol). Diabetes duration was compared using Fisher’s exact test.

Variable	Active (*n* = 13)	Sedentary (*n* = 10)	*p*-Value *
Sex:			0.384
M	4	5	
F	9	5	
Age (years)	57.5 ± 10.4	64.4 ± 5.2	0.093
Height (cm)	164.7 ± 10.1	168.8 ± 7.6	0.416
Weight (kg)	80.8 ± 19.9	94.2 ± 17.6	0.160
BMI (kg/m^2^)	29.5 ± 5.2	33.0 ± 5.6	0.194
Abdominal circumference (cm)	103.7 ± 15.5	115.3 ± 13.7	0.169
HbA1c (mmol/mol)	57.0 ± 20.3	55.4 ± 12.0	0.913
Fasting glucose (mg/dL)	157.6 ± 73.0	142.9 ± 60.6	0.777
Diabetes duration:			1.000
<5 years	5	3	
≥5 years	8	7	

* One-way ANOVA, except for Sex (Fisher’s exact test).

**Table 2:** Summary of repeated-measures ANOVA findings for stabilometric and EMG outcomes. Results summarize main effects and interactions reported in the Section 3. n.s. = non-significant (*p* > 0.05).

Outcome	Effect	F	*p*	ηp^2^
ML COP	Stimulus	2.934	0.045	0.557
ML COP	Side × time × group × sex	4.430	0.027	0.330
ML COP	Group	n.s.	—	—
AP COP	Stimulus	3.47	0.026	0.598
AP COP	Group	n.s.	—	—
COP speed	Stimulus	26.43	<0.001	0.919
COP speed	Time × sex	4.05	0.035	0.311
COP speed	Group	n.s.	—	—
SOL EMG	Stimulus	9.16	<0.001	0.753
TA EMG	Side × time × group	3.55	0.050	0.283

## Data Availability

The data that support the findings of this study are not openly available due to reasons of sensitivity and are available from the corresponding author upon reasonable request. Data are located in controlled access data storage at the University of Bologna (Italy).
